# Modulation of IL-17A and TL1A largely abrogates house dust mite-induced lung inflammation in murine model of allergic airway disease

**DOI:** 10.1186/1476-9255-10-S1-P3

**Published:** 2013-08-14

**Authors:** C Hubeau, J Kubera, K Hammerman, J Wright J, M Denz, Y-T Juang, C Williams

**Affiliations:** 1 Inflammation and Remodeling, Pfizer, Cambridge, Massachusetts, 02140, USA; 2 Drug Safety R&D, Pfizer, Cambridge, Massachusetts, 02140, USA; 3 Immunoscience, Pfizer, Cambridge, Massachusetts, 02140, USA

## 

Recent studies suggest a role for Th17 responses in the increased airway neutrophilia associated with severe asthma. House dust mite (HDM) is a natural allergen to which asthmatics are often sensitized. Mice repeatedly challenged with HDM extract developed robust airway neutrophilia rapidly evolving into asthma-like disease with increased numbers of eosinophils and lymphocytes in bronchoalveolar lavages (BAL) as well as inflammatory infiltrates, vascular/muscular hypertrophy, interstitial fibrosis, epithelial hyperplasia and mucus accumulation in lung tissues. RNA and protein screening revealed a robust Th17 component post-HDM exposure. We thus evaluated whether IL-17A deficiency could modulate HDM-induced allergic airway disease. Airway neutrophilia was indeed abrogated in IL-17A deficient mice weekly challenged with HDM (acute model), however total BAL cellularity and lung mechanics remained comparable to those of HDM-challenged WT mice. In contrast, IL-17A deficient mice daily exposed to HDM (chronic model) had decreased BAL cellularity associated with reduced numbers of BAL macrophages, neutrophils, eosinophils and lymphocytes. Interestingly antibody neutralization of TL1A, a member of the TNF superfamily known to promote Th2 and Th17 responses, reduced BAL cellularity to baseline levels in HDM-challenged WT mice. Our results thus indicate that targeting Th17 responses can alleviate HDM-induced airway neutrophilia, and can also broadly modulate allergic airway disease.

